# Management Patterns of Croup in Korean Emergency Departments: A Nationwide Cohort Study

**DOI:** 10.3390/children12101301

**Published:** 2025-09-25

**Authors:** Jin Hee Kim, Jae Yun Jung, Soyun Hwang, Joong Wan Park, Eui Jun Lee, Ha Ni Lee, Do Kyun Kim, Young Ho Kwak

**Affiliations:** 1 Department of Emergency Medicine, CHA Bundang Medical Center, CHA University School of Medicine, Seongnam 13496, Korea; heartfeltdoctor@chamc.co.kr; 2 Department of Emergency Medicine, Seoul National University Hospital, 101 Daehak-ro, Jongno-gu, Seoul 03080, Korea; zzibii17@snu.ac.kr (J.W.P.); eklesia7@snu.ac.kr (E.J.L.); bird001@snu.ac.kr (D.K.K.); yhkwak@snuh.org (Y.H.K.); 3 Department of Pediatrics, Severance Children’s Hospital, 50-1 Yonsei-ro, Seodaemun-gu, Seoul 03722, Korea; hani1109@yuhs.ac (H.N.L.)

**Keywords:** croup, emergency departments, children, steroids

## Abstract

**Highlights:**

**What are the main findings?**

- Despite guideline recommendations, only around half of children with croup in Korean emergency departments received corticosteroids.
- Dedicated pediatric emergency centers (DPECs) were associated with lower use of potentially low-value interventions (salbutamol and chest and cervical radiography) than general emergency centers (GECs).

**What is the implication of the main finding?**

- Education and implementation of standardized national clinical guidelines are needed to optimize croup management in Korean emergency departments.

**Abstract:**

**Background:** Despite the established importance of prescribing steroids to children with croup, many physicians in Korean emergency departments (EDs) do not adhere to this recommendation. This study aimed to evaluate treatment appropriateness by investigating steroid prescription rates and potentially low-value interventions such as salbutamol nebulizers and radiographs and to compare dedicated pediatric emergency centers (DPECs) and general emergency centers (GECs) to understand treatment trends for croup in Korea. **Methods:** This retrospective cohort study analyzed a 5% random sample of the National Health Screening Program for Infants and Children (NHSPIC) cohort linked to the National Health Insurance Service database (2008–2015). The study included children with a primary diagnosis of croup and excluded children who were prescribed oral or steroid injections within three days before their ED visit. The primary outcome was steroid prescription rate; secondary outcomes included comparisons of management patterns between DPECs and GECs. **Results:** The overall steroid prescription rate was 56.9%. Steroid prescribing was slightly higher in DPECs than in GECs (61.2% vs. 56.3%, *p* = 0.131). In contrast, DPECs had lower prescription rates for salbutamol nebulizers (4.5% vs. 12.7%, *p* < 0.001), chest radiographs (65.3% vs. 78.7%, *p* < 0.001), and cervical spine radiographs (4.5% vs. 12.6%, *p* < 0.001). Steroid prescription rates showed no significant temporal trend, while potentially low-value interventions decreased significantly. **Conclusions:** Only about half of children with croup in Korean EDs received steroids. DPECs were associated with lower use of potentially low-value interventions, suggesting more guideline-concordant practice. Education and implementation of standardized national croup clinical guidelines are needed to optimize care.

## 1. Background

Croup is a common cause of pediatric emergency department (ED) visits, characterized by stridor on inspiration, a characteristic barking cough, and hoarseness due to edema and inflammation in the upper airway [1,2]. The prevalence of croup in 1-year-olds is 5% per year, and it affects 3% of all children every year [2,3]. According to a review article in 2018, approximately 60% of croup patients had a mild spontaneous course of symptom improvement within 48 h [4]. Approximately 22.8% of croup patients visiting emergency departments require hospitalization, and the mortality rate is less than 0.5%, which is very rare in Korea [5].

Careful medical history and physical examinations are the best methods for confirming a croup diagnosis [4]. The medical treatments recommended for croup are mainly steroids and an epinephrine nebulizer [2,6]. Regardless of severity, steroids are routinely recommended for mild to severe croup because they effectively relieve symptoms within two hours, shorten hospital stays, and lower the rate of patient revisits or re-admissions [7]. A previous U.S. study comparing the effects of dexamethasone and prednisone for about 6 years in patients with hospitalized croup through ED revisit or re-admission rates within 7 days also found that both corticosteroids were reasonable choices for treating croup [8]. However, among steroids, dexamethasone can be administered orally or intramuscularly, and it is particularly favored because it has an easier route of administration. It also has the advantage of a single dose being sufficient because of its long-lasting action. In contrast, epinephrine nebulization is the recommended treatment for moderate-to-severe croup [6,9,10]. However, despite the established treatment guidelines, there is a lack of consistency regarding treatment choice among hospitals in actual practice [2,6,9,10]. In a study conducted in EDs in Canada, there were significant differences in the management of uncomplicated croup between general emergency physicians and pediatricians. Emergency physicians were more likely than pediatricians to prescribe chest radiographs, racemic epinephrine, salbutamol, and parenteral steroids to their patients in ED [11].

According to an analysis of the 2005 National Hospital Ambulatory Medical Care Survey (NHAMCS) in the U.S., only 31% of croup patients were prescribed steroids, even though steroids were the recommended treatment for croup, regardless of severity [12]. In another study using the same database, the steroid prescription rate increased to 56% in 2009, and an analysis of 2010–2015 data showed that the steroid prescription rate for croup in U.S. EDs increased to over 70% [13]. However, only 63.8% of patients were prescribed any form of corticosteroids in the retrospective study that analyzed treatment patterns and adherence to recommendations for croup in two Italian pediatric EDs in 2017 [14]. In addition, although not statistically significant, steroid prescription rates were higher in dedicated pediatric emergency centers (DPECs) than in general emergency centers (GECs) [15]. Moreover, a previous study showed that the management of croup cases varies depending on the physician’s specialty. Compared with physicians with a pediatric background, emergency medicine-trained physicians who managed uncomplicated cases of croup were more likely to conduct radiographs and prescribe racemic epinephrine, salbutamol, and parenteral steroids in the ED [11].

In Korea, according to our knowledge, no study has specifically evaluated the appropriateness of steroid prescriptions for pediatric croup in emergency departments or compared management between DPECs and GECs using nationwide data. Therefore, we analyzed whether croup patients visiting Korean EDs receive appropriate treatment using cohort data from the National Health Screening Program for Infants and Children (NHSPIC) [16]. The primary aim of this study was to evaluate the appropriateness of croup management in Korean EDs, particularly focusing on the use of steroids. The secondary aim was to compare prescription patterns and the use of potentially low-value interventions, including salbutamol nebulizers and radiographs, between DPECs and GECs.

## 2. Methods

### 2.1. Study Setting and Data Sources

The NHSPIC cohort is a population-based sample established by the National Health Insurance Service of Korea. It consists of approximately 5% of all children born between 2008 and 2012 who underwent at least the first or second round of the national infant health screening program, with linked health insurance claims data. The Korean government launched the NHSPIC, a population surveillance system, in November 2007. It was developed for the evaluation of the growth and development of infants and children, early detection of disease, and provision of guidance for predictable disease and injury prevention according to age. Seven NHSPIC screening surveys were conducted on all children from the age of 4 months to 71 months. The health checkups scheduled were as follows: first, age 4–6 months; second, age 9–12 months; third, age 18–24 months; fourth, age 30–36 months; fifth, 42–48 months; sixth, 54–60 months; seventh, age 66–71 months [17,18]. We used data established between 2008 and 2015 (8 years), which provides a 5% random sample of the population of children born from 1 January 2008, to 31 December 2012, who completed the first or second session of the NHSPIC (n = 83,910) [19].

This cohort database contains information on demographic characteristics, including age, sex, medical insurance status, region, and date of death. Insurance type was categorized according to the household head (self-employed, employee, or medical aid), thereby approximately reflecting the socioeconomic environment of the child’s family. It also provides information on the results of the National Health Screening Program and health insurance claim data for every healthcare visit during the study period. Healthcare utilization data were collected when an individual received healthcare services; these data included: date of visit, type of medical institution (clinic/hospital/tertiary hospitals/public health center), and type of visit (inpatient/outpatient/emergency/intensive care). The healthcare provider data contains information about location, type of hospital, number of beds, medical equipment, human resources, and physicians’ specialties. We extracted data on the location and physician’s specialty for our study. Medical records included the International Classification of Diseases-10 (ICD-10) code, length of hospital stay, medical services received, and prescription records (drug classification code, days prescribed) [17].

### 2.2. Study Population

**Figure 1:**
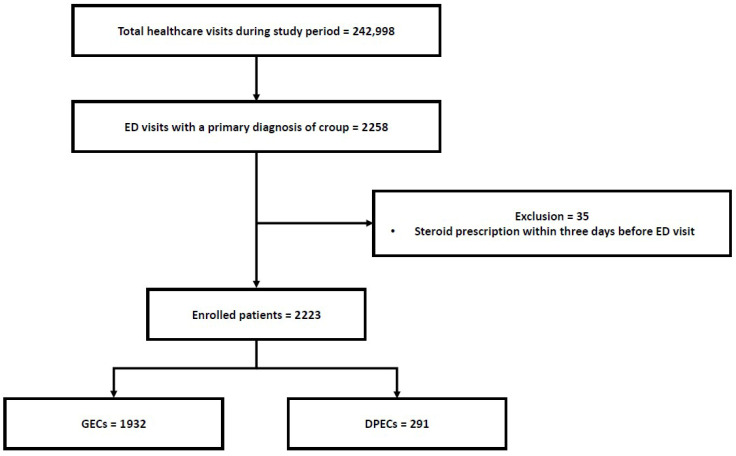
The study population. ED, emergency department; GECs, general emergency centers; DPECs, dedicated pediatric emergency centers.

The study population is shown in Figure 1. Among the 242,998 healthcare visits made by the cohort participants, we included patients with croup (ICD-10 code J05.0) recorded as the primary diagnosis for the ED visit. Visits in which J05.0 appeared only as a secondary diagnosis were excluded to ensure that the study population represented children whose main reason for the ED visit was croup. We excluded children who had been prescribed oral or steroid injections within three days before their ED visits, as dexamethasone—the long-acting corticosteroid most frequently administered—has an effect lasting up to 72 h.

### 2.3. Dedicated Pediatric Emergency Centers (DPECs) in Korea

DPECs opened in Korea in 2010 and were formally designated by the Ministry of Health and Welfare of Korea in 2011. Initially, four hospitals were designated, and additional hospitals were added in subsequent years as they met the required criteria, bringing the total number of participating hospitals to 10 during our study period. Based on official records and our dataset, six hospitals (A–F) were designated in 2011, followed by two hospitals (H and I) in 2013, and two more hospitals (G and J) in 2014. However, hospitals that did not meet the criteria were subsequently disqualified [20,21,22]. Importantly, all 10 DPECs maintained their designation status through the end of our study period in 2015. Finally, as of December 2022, eight hospitals were operating under the DPEC program [23].

### 2.4. Variables and Measurement

We collected data on demographic variables, including age, sex, regional distribution, and type of national health insurance. It does not provide the exact date of birth; each child’s age was grouped by year. We subdivided the patients into eight groups by year. For example, if a child was born in 2008, the child was categorized as Group 0 (0-year-old) in 2008 and as Group 1 (1-year-old) on 1 January 2009. Regarding regional distribution, those who lived in Seoul and six metropolitan cities (Busan, Daegu, Incheon, Gwangju, Daejeon, and Ulsan) were defined as “urban” and other areas as “rural” [17]. Types of national health insurance in Korea were classified as either self-employed, employee, or medical aid beneficiary.

To investigate proper croup management according to guidelines during the study period and to confirm the difference between DPECs and GECs from 2008 to 2015, we collected treatment-related variables, including prescription of steroids, epinephrine nebulizer from services received, and prescription records. To show which types of steroids were most prescribed, we categorized prescribed steroids (dexamethasone, prednisolone, hydrocortisone, and methylprednisolone). We did not separately distinguish the route of administration of the treatments because there is a lack of evidence that the therapeutic effect of steroids differs depending on the route of administration according to the latest guidelines [14]. However, detailed information on dosing was not available in the claims database. To identify inappropriate treatments and tests that did not follow the guidelines, we also collected the most representative inappropriate treatment, including salbutamol nebulizer and potentially low-diagnostic yield tests (chest radiographs and cervical spine radiographs), as variables [2,6,9,10,11].

We investigated physicians’ specialty in EDs at discharge to show the differences in croup management related to physicians’ specialty for both trainees and attending physicians, including emergency medicine (EM), pediatrics (PED), surgery, internal medicine, otolaryngology, and family medicine specialists. We additionally examined the outcomes of emergency department (ED) visits. In Korean insurance claims data, an ED stay ≥6 h is defined as hospitalization; thus, we classified <6 h as discharge, acknowledging that this claims-based definition may not accurately represent patients’ actual disposition.

### 2.5. Outcomes

The primary outcome of this study was to investigate the steroid prescription rate for croup treatment in Korean EDs. The secondary outcomes involved comparing steroid prescriptions between DPECs and GECs and evaluating potentially low-value interventions such as nebulizer use and radiographs.

### 2.6. Statistical Analysis

**Figure 2:**
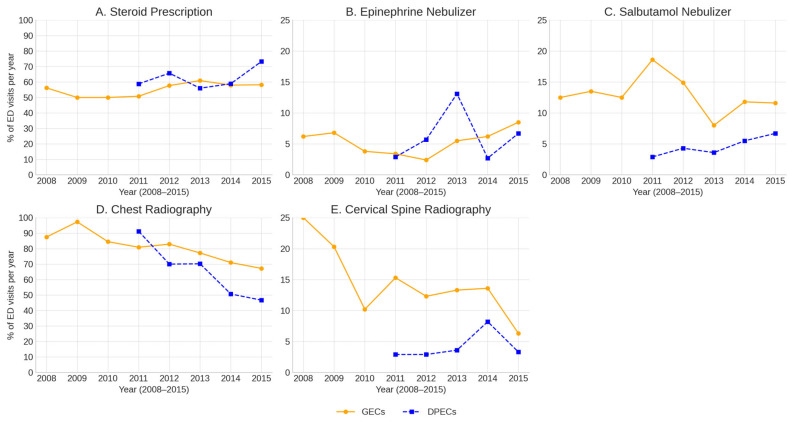
Annual trends in management patterns of croup (2008–2015), stratified by center type. (**A**) Total steroid prescriptions; (**B**) epinephrine nebulizer use; (**C**) salbutamol nebulizer use; (**D**) chest radiography; (**E**) cervical spine radiography. Percentages represent the proportion of ED visits for croup in each year. Solid line with circles = GECs; dashed line with squares = DPECs; ED, emergency department; GECs, general emergency centers; DPECs, dedicated pediatric emergency centers.

All statistical analyses were performed using R-packages version 4.0.2 (R Foundation for Statistical Computing, Vienna, Austria). After testing for normality, proportions were calculated for categorical variables. Differences in categorical variables were assessed using the chi-squared test, and differences in continuous variables were assessed using the Wilcoxon rank-sum test. To evaluate the annual trends of management patterns of croup, linear regression analysis was used with the year as a continuous variable. Risk differences (RDs) and 95% confidence intervals (CIs) were also calculated to quantify absolute differences between GECs and DPECs. We used unadjusted RDs because this study was designed as descriptive surveillance based on claims data. The significance level was set at 0.05. Figure 2 illustrating annual trends was generated using Python version 3.11.8 (Python Software Foundation, Wilmington, DE, USA) with the matplotlib (version 3.6.3) and seaborn (version 0.11.2) packages.

### 2.7. Ethics

This study was approved by the Institutional Review Board of Seoul National University Hospital (No. E-2108-003-1240).

## 3. Results

### 3.1. Characteristics of the Study Population

Figure 1 presents a flowchart depicting this study. A total of 2258 ED visits were made by infants and children with croup during the study period. After excluding cases where steroids were prescribed before three days of the ED visit, 2223 cases were included in the final analysis (1932 for GECs and 291 for DPECs). There were no cases of cardiopulmonary resuscitation or endotracheal intubation for breathing support during the visits. Of the total visits, 33.6% of the patients were female (34.3% in GECs and 29.2% in DPECs), and the peak age group was Group 2, which was children born from 1 January 2010 to 31 December 2010. Regarding regional distribution, 1262 (56.8%) of the visits were in rural areas. There were no significant between-group differences in sex and type of national health insurance (Table 1).

### 3.2. Primary Outcome

Based on the cohorts in the NHSPIC from 2008 to 2015, the steroid prescription rate for children who visited EDs with a diagnosis of croup was 56.9%. There was no statistically significant difference in steroid prescription rates between DPECs and GECs (61.2% and 56.3%, respectively; *p* = 0.131, Table 2).

### 3.3. Secondary Outcomes

The overall epinephrine nebulizer prescription rate was 5.1%, and there was no statistically significant difference between DPECs and GECs (*p* = 0.178, Table 2). During the study period, the total salbutamol nebulizer prescription rate for children with croup was 11.6%. The prescription rates for salbutamol nebulizers in DPECs and GECs were 4.5% and 12.7%, respectively; these were significantly lower in DPECs (*p* < 0.001, Table 2). The overall rates of low-diagnostic yield testing were 76.9% for chest radiographs and 11.6% for cervical spine radiographs, with significantly lower rates observed in DPECs (*p* < 0.001, Table 2).

Among the physicians’ specialties, EM (51.8%) and PED (47.9%) were similarly distributed in DPECs (51.2% and 48.8%, respectively) and GECs (51.9% and 47.7%, respectively), with no statistically significant differences (*p* = 0.950, Table 2). Regarding the ED visits of infants and children with croup, 1563 (70.3%) patients were discharged. The discharge rate was significantly higher in DPECs than in GECs (85.2% and 68.1%, *p* < 0.001, Table 2).

Analyzing the change in the steroid prescription rate in EDs over the years reveals no statistically significant change (56.2% in 2008 to 60.3% in 2015; *p* for trend = 0.064). During the study period, we observed a statistically significant decrease in the prescription rate of salbutamol nebulizers, which decreased from 12.5% to 11.0% (*p* for trend = 0.012, Table 3). However, there was no significant change in the prescription rate of salbutamol nebulizers in DPECs. The rates for the chest (87.5% to 64.6%, *p* for trend < 0.001) and cervical spine radiographs (25.0% to 5.9%, *p* for trend = 0.015) also significantly decreased (Table 3). The prescription rate of chest radiography in DPECs significantly decreased from 91.2% to 46.7% (*p* for trend < 0.001, Table 3). However, there was no significant change in the rate of cervical spine radiography in DPECs. Figure 2 shows the annual trends in management patterns of croup, stratified by center type.

The detailed prescription status for each steroid type is summarized in Table S1. Dexamethasone was the most prescribed steroid, with a higher prescription rate in DPECs than in GECs. Prednisolone, hydrocortisone, and methylprednisolone were also prescribed. We analyzed various steroids, including triamcinolone, betamethasone, and deflazacort, but there were no cases other than the four steroids mentioned in Table S1. It is important to note that this table takes into account cases where there have been multiple prescriptions of steroids to the same child. Additional detailed results are provided in Tables S1–S4 for reference.

## 4. Discussion

Regardless of severity, steroids are an essential treatment for children with croup. Nevertheless, according to our results, derived from the NHSPIC from 2008 to 2015, the overall steroid prescription rate for croup patients was approximately 56.9%. Compared with the analysis of 2010–2015 NHAMCS data, the steroid prescription rate for croup in U.S. EDs increased from 56% in 2009 to over 70% in 2015 [12].

In this study, the overall steroid prescription rates in DPECs and GECs were 61.2% and 56.3%, respectively. This difference was not statistically significant. According to a U.S. study that attempted to measure differences in emergency care for children between GECs and DPECs from 2010 to 2015, no differences in the performance of GECs and DPECs were found for corticosteroids for croup (+4.1%, 95% CI = –7.2 to 15.5), similar to the previous study [15]. Although our study found no overall difference between GECs and DPECs, the markedly low steroid prescription rate at one DPEC (Hospital D) likely obscured differences, as shown in Table S2. Excluding this outlier, DPECs had significantly higher steroid use than GECs (71.8% vs. 56.3%, *p* < 0.001; Table S3). As most DPECs in Korea have continued to operate stably since their designation, future analyses of more recent data will be important to determine whether steroid prescribing for pediatric croup has improved.

Although the difference in the total steroid prescription rates in the DPECs was not statistically significant, we noted a gradual increase in the rate from 58.8% in 2011 to 73.3% in 2015 (Table 3 and Table S4). According to a population-based sample survey conducted to evaluate adherence to guidelines in the management of pediatric croup in Australia in 2019, most children received adequate treatment and did not undergo unnecessary tests, unlike in our findings [24]. However, as mentioned for two Italian pediatric EDs, 403/632 (63.8%) children were prescribed corticosteroids in a 2017 retrospective study that analyzed treatment patterns and adherence to recommendations for croup patients [14]. This was similar to the results of our steroid prescription rate in DPECs.

During the study period, the overall prescription rates of salbutamol nebulizers in DPECs and GECs were 4.5% and 12.7%, respectively. The significantly lower prescription rate of salbutamol nebulizers in DPECs than in GECs indicates that DPECs were associated with lower use of potentially low-value treatment. Consistent with our findings, a retrospective study of practice variation reported higher bronchodilator use among EM-trained physicians compared with pediatric providers (PED cohort 4.4%, Pediatric EM cohort 5.4%, and EM cohort 14%, *p* < 0.01) [11]. The higher salbutamol prescription rate in GECs likely reflects greater diagnostic uncertainty, as children with croup are sometimes concomitantly coded with bronchiolitis when wheezing is present. Notably, however, DPECs in our cohort had more frequent co-diagnoses of bronchiolitis, suggesting that case mix alone cannot explain the discrepancy. Instead, the findings more plausibly reflect variation in adherence to guideline-recommended croup management.

Although a statistically lower proportion of radiographs were used in DPECs, chest radiographs were used by most providers in both DPECs and GECs (65.3% and 78.7%, respectively). The excessive use of radiographs may have been due not only to a lack of clinical confidence in decision-making for croup-like diseases in Korea, but also to concomitant clinical suspicion of pneumonia, as 4.7% of our cohort was coded with unspecified pneumonia. Although antibiotic prescriptions were not specifically analyzed, this diagnostic overlap may have contributed to the higher utilization of chest radiographs compared with the 17% previously reported [11]. However, our results in Table 3 showed a significant annual decrease in chest radiographs, with only 46.7% of patients in DPECs receiving them in 2015. In contrast, the use of cervical spine radiography in DPECs was significantly lower, with only 4.5% of patients. This finding is consistent with a previous study cited in this paper [11], which reported that lateral neck radiographs were performed in less than 10% of cases, similar to our study. Notably, except for one DPEC in Table S4, the cervical spine radiograph was not performed at all in other DPECs. Although our study was a sample cohort study, it is significant to report that DPECs rarely performed cervical radiological studies to diagnose croup. Croup is a clinical diagnosis in most cases, whereas other respiratory diseases in children and adolescents often require radiological or microbiological test for confirmation. Although radiographs can be performed to differentiate other diseases that can cause acute upper airway obstruction, it was not possible to determine whether such indications were present, as our database did not include detailed medical history and physical examination findings. Although Korean guidelines for antibiotic use in children with acute upper respiratory tract infections suggest that a lateral cervical radiograph may be used to confirm the thumbprint sign, although these characteristic findings are not always present in all croup cases [25]. Therefore, the routine use of chest or cervical spine radiography in croup patients should be avoided [11].

Overall, ED discharge was 70.3%, significantly higher in DPECs than in GECs. This finding may reflect differences in the experience or practice style of physicians in DPECs. However, as our dataset lacked measures of disease severity, caution should be exercised when interpreting higher discharge rates. Contrary to our results, the ultimate disposition (e.g., discharge medications and admission rate) of croup did not differ significantly in a retrospective comparative study of practice variation between EM and pediatric physician. However, as the same study showed that the mean length of ED stay (minutes) and direct hospital costs were significantly higher in the EM cohort, it would be important to compare the length of stay and cost if future studies were conducted [11].

Our secondary outcomes also highlighted that potentially low-value interventions were still used in the management of croup in both DPECs and GECs. A recent study emphasized that the de-implementation of low-value interventions is important for improving overall health outcomes and reducing healthcare costs [26]. Therefore, high-quality randomized controlled trials are needed in the future to show the need to decrease de-implementation interventions and increase adherence to treatment guidelines for croup.

To our knowledge, this is the first study to investigate the management patterns of croup in Korean EDs using a nationwide infant cohort sample, and the first to compare practice differences between DPECs and GECs. While this sample cohort does not represent all children in Korea and may have inherent selection limitations, its randomized and population-based design still provides meaningful insights into treatment behaviors across diverse emergency settings. Unlike prior single-center reports, our study offers broader generalizability and provides an important foundation for future nationwide investigations with more comprehensive datasets [27,28].

It is imperative that guidelines and educational resources regarding croup care standards are needed. A comparative study conducted before and after the implementation of the croup clinical pathway in Australian EDs supports this. [29]. Improving adherence to the recommendations for using corticosteroids in croup leads to clinical and economic benefits [14]. Additionally, targeted interventions, coupled with the implementation of low-value care can be instrumental in promoting the effective implementation of standard guidelines [30].

### Limitations

This study has some limitations. First, this is a retrospective cross-sectional study based on infant cohort data from the NHSPIC. As in a previous study on croup in Korea using the National Emergency Department Information System (NEDIS), the database used in our study did not contain detailed history, physical examination findings, and laboratory findings [5]. Furthermore, since the database was anonymized at the visit level, we were unable to track long-term prognosis of individual patients or account for repeated visits by the same child, which may have inflated the effective sample size. Future studies using datasets that allow patient-level linkage will be necessary to validate our findings. Additionally, we could not exclude patients with congenital anomalies of the respiratory system or chronic respiratory diseases from the study population. Nevertheless, this cohort for infants and children is useful for analyzing the impact of infantile disease outcomes, nutritional status, and safety education [17].

Second, the study population did not include all infants and children in Korea during the study period, and while croup is most commonly observed between 6 months and 3 years of age, our cohort encompassed a wider age range up to 8 years. According to the 2015 Annals of National Health Examination Statistics issued by the National Health Insurance Corporation in Korea, the screening rates for infants and young children were 50.1% in 2010, 69.8% in 2014, and 69.5% in 2015. Although our cohort included individuals up to 8 years of age, as indicated in Table 1, the proportion of cases beyond the age range of typical croup occurrence was relatively low. Despite this limitation, the study had a representative sample cohort [17]. Moreover, the study was significant in that the sample size was large in the research-related croup of ED, and it was analyzed separately from DPECs and GECs.

Third, although we evaluated and compared the prescription rate of epinephrine nebulizer use, disease severity could not be inferred from this database. Validated severity scales, such as the Westley croup score, were unavailable, and treatment decisions may have been influenced by unmeasured clinical factors, raising the possibility of confounding by indication. Furthermore, although we excluded children who had received corticosteroids within three days prior to the ED visit to reduce bias from pre-treatment, the exact dosing and timing of steroid administration during the ED visit could not be determined from the claims data. This limited our ability to fully assess adherence to guideline-recommended steroid regimens. In addition, provider specialty information may have been misclassified in the claims data, which could have influenced the observed differences between DPECs and GECs.

Finally, our analyses were limited to unadjusted comparisons, and future studies should use risk-adjusted or multi-level models to address confounding and clustering. In addition, potential misclassification between GECs and DPECs over time cannot be excluded; however, according to official records, all 10 designated DPECs maintained their status through the end of our study period, which may lessen the risk of such misclassification.

## 5. Conclusions

Using a nationwide cohort from the NHSPIC, we found that only 56.9% of patients with croup presenting to Korean EDs received steroids, indicating that prescribing practices remain suboptimal. Although DPECs were associated with lower use of salbutamol and radiography compared with GECs, substantial variation in management persisted. These findings underscore the urgent need for standardized national clinical guidelines, targeted physician education, and efforts to reduce potentially low-value interventions.

## Figures and Tables

**Table 1:** Demographic characteristics of cohort participants.

Variables	Overall (N = 2223)	GECs (N = 1932)	DPECs (N = 291)	*p*-Value
Gender				0.098
Female	748 (33.6)	663 (34.3)	85 (29.2)	
Age group (Year of birth)				0.009
Group 0 (2008)	105 (4.7)	102 (5.3)	4 (1.4)	
Group 1 (2009)	565 (25.4)	488 (25.3)	77 (26.5)	
Group 2 (2010)	739 (33.2)	657 (34.0)	82 (28.2)	
Group 3 (2011)	463 (20.8)	388 (20.1)	75 (25.8)	
Group 4 (2012)	208 (9.4)	173 (9.0)	35 (12.0)	
Group 5 (2013)	98 (4.4)	87 (4.5)	11 (3.8)	
Group 6 (2014)	36 (1.6)	31 (1.6)	5 (1.7)	
Group 7 (2015)	8 (0.4)	6 (0.3)	2 (0.7)	
Region				0.002
Rural area	1262 (56.8)	1122 (58.1)	140 (48.1)	
Type of national health insurance				0.112
Self-employed	554 (24.9)	495 (25.6)	59 (20.3)	
Employee	1597 (71.8)	1373 (71.1)	224 (77.0)	
Medical aid	72 (3.2)	64 (3.3)	8 (2.7)	

Values are presented as number (% of croup ED visits); ED, emergency department; GECs general emergency centers; DPECs, dedicated pediatric emergency centers.

**Table 2:** Primary and secondary outcomes.

Variables	Overall (N = 2223)	GECs (N = 1932)	DPECs (N = 291)	Risk difference (95% CI)	*p*-Value
Total steroid prescription	1265 (56.9)	1087 (56.3)	178 (61.2)	4.9% (−3.0–12.5)	0.131
Nebulizer					
Epinephrine nebulizer	113 (5.1)	93 (4.8)	20 (6.9)	2.1% (−1.4–6.4)	0.178
Salbutamol nebulizer	258 (11.6)	245 (12.7)	13 (4.5)	−8.2% (−11.6–−3.8)	<0.001
Radiographs					
Chest radiographs	1710 (76.9)	1520 (78.7)	190 (65.3)	−13.4% (−20.8–−6.3)	<0.001
Cervical spine radiographs	257 (11.6)	244 (12.6)	13 (4.5)	−8.2% (−11.6–−3.7)	<0.001
Sub-diagnosis respiratory disease					0.346
Viral pneumonia (J12)	1 (0.0)	1 (0.1)	0 (0.0)	−0.1% (−0.3–1.3)	
Bacterial pneumonia (J15)	3 (0.1)	3 (0.2)	0 (0.0)	−0.2% (−0.5–1.3)	
Pneumonia, unspecified (J18)	104 (4.7)	97 (5.0)	7 (2.4)	−2.6% (−4.9–0.7)	
Acute bronchitis (J20)	302 (13.6)	260 (13.5)	42 (14.4)	1.0% (−4.2–6.9)	
Acute bronchiolitis (J21)	180 (8.1)	152 (7.9)	28 (9.6)	1.8% (−2.4–6.8)	
Acute lower respiratory infection, unspecified (J22)	1 (0.0)	1 (0.1)	0 (0.0)	−0.1% (−0.3–1.3)	
No sub-diagnosis	1632 (73.4)	1418 (73.4)	214 (73.5)	0.1% (−7.1–6.9)	
Physician’s specialty					0.950
Emergency medicine	1152 (51.8)	1003 (51.9)	149 (51.2)	−0.7% (−8.7–7.2)	
Pediatrics	1064 (47.9)	922 (47.7)	142 (48.8)	1.1% (−6.8–9.0)	
Surgery	4 (0.2)	4 (0.2)	0 (0.0)	−0.2% (−0.5–1.2)	
Internal medicine	1 (0.0)	1 (0.1)	0 (0.0)	−0.1% (−0.3–1.3)	
Otolaryngology	1 (0.0)	1 (0.1)	0 (0.0)	−0.1% (−0.3–1.3)	
Family medicine	1 (0.0)	1 (0.1)	0 (0.0)	−0.1% (−0.3–1.3)	
ED outcome					
Discharge (ED stay < 6 h)	1563 (70.3)	1315 (68.1)	248 (85.2)	17.2% (10.6–22.9)	<0.001

Values are presented as number (% of croup ED visits); ED, emergency department; GECs general emergency centers; DPECs, dedicated pediatric emergency centers; RD, risk difference; CI, confidence interval.

**Table 3:** Changes in ED prescription drugs and radiological examinations for croup by year.

Variables		2008	2009	2010	2011	2012	2013	2014	2015	*p*-Value
Overall	ED visits (N = 2223)	16	74	264	270	486	483	411	219	
	Total steroid prescription	9 (56.2)	37 (50.0)	132 (50.0)	140 (51.9)	286 (58.8)	290 (60.0)	239 (58.2)	132 (60.3)	0.064
	Dexamethasone	7 (43.8)	35 (47.3)	105 (39.8)	124 (45.9)	241 (49.6)	227 (47.0)	191 (46.5)	114 (52.1)	0.230
	Epinephrine nebulizer	1 (6.2)	5 (6.8)	10 (3.8)	9 (3.3)	14 (2.9)	33 (6.8)	23 (5.6)	18 (8.2)	0.028
	Salbutamol nebulizer	2 (12.5)	10 (13.5)	33 (12.5)	45 (16.7)	65 (13.4)	35 (7.2)	44 (10.7)	24 (11.0)	0.012
	Chest radiography	14 (87.5)	72 (97.3)	223 (84.5)	222 (82.2)	394 (81.1)	367 (76.0)	277 (67.4)	141 (64.4)	<0.001
	Cervical spine radiography	4 (25.0)	15 (20.3)	27 (10.2)	37 (13.7)	53 (10.9)	56 (11.6)	52 (12.7)	13 (5.9)	0.015
GECs	ED visits (N = 1932)	16	74	264	236	416	399	338	189	
	Total steroid prescription	9 (56.2)	37 (50.0)	132 (50.0)	120 (50.8)	240 (57.7)	243 (60.9)	196 (58.0)	110 (58.2)	0.079
	Dexamethasone	7 (43.8)	35 (47.3)	105 (39.8)	109 (46.2)	204 (49.0)	185 (46.4)	153 (45.3)	95 (50.3)	0.409
	Epinephrine nebulizer	1 (6.2)	5 (6.8)	10 (3.8)	8 (3.4)	10 (2.4)	22 (5.5)	21 (6.2)	16 (8.5)	0.036
	Salbutamol nebulizer	2 (12.5)	10 (13.5)	33 (12.5)	44 (18.6)	62 (14.9)	32 (8.0)	40 (11.8)	22 (11.6)	0.013
	Chest radiography	14 (87.5)	72 (97.3)	223 (84.5)	191 (80.9)	345 (82.9)	308 (77.2)	240 (71.0)	127(67.2)	<0.001
	Cervical spine radiography	4 (25.0)	15 (20.3)	27 (10.2)	36 (15.3)	51 (12.3)	53 (13.3)	46 (13.6)	12 (6.3)	0.023
DPECs	ED visits (N = 291)	N/A	N/A	N/A	34	70	84	73	30	
	Total steroid prescription	N/A	N/A	N/A	20 (58.8)	46 (65.7)	47 (56.0)	43 (58.9)	22 (73.3)	0.452
	Dexamethasone	N/A	N/A	N/A	15 (44.1)	37 (52.9)	42 (50.0)	38 (52.1)	19 (63.3)	0.637
	Epinephrine nebulizer	N/A	N/A	N/A	1 (2.9)	4 (5.7)	11 (13.1)	2 (2.7)	2 (6.7)	0.092
	Salbutamol nebulizer	N/A	N/A	N/A	1 (2.9)	3 (4.3)	3 (3.6)	4 (5.5)	2 (6.7)	0.930
	Chest radiography	N/A	N/A	N/A	31 (91.2)	49 (70.0)	59 (70.2)	37 (50.7)	14 (46.7)	<0.001
	Cervical spine radiography	N/A	N/A	N/A	1 (2.9)	2 (2.9)	3 (3.6)	6 (8.2)	1 (3.3)	0.514

Values are presented as number (% of croup ED visits for each year). ED, emergency department; GECs general emergency centers; DPECs, dedicated pediatric emergency centers; N/A, not applicable (not yet designated as DPEC).

## Data Availability

Restrictions apply to the availability of these data. The data were obtained from the National Health Insurance Service (NHIS) database of Korea; however, the raw data are no longer publicly available and cannot be obtained from the authors or the NHIS.

## Supplementary Materials

The following supporting information can be downloaded at: https://www.mdpi.com/article/10.3390/children12101301/s1, Table S1: Steroids in detail. Table S2: Changes in total steroid prescription by year in DPEC. Table S3: Primary and secondary outcomes, excluding 1 DPEC. Table S4: Changes in ED prescription drugs and radiological examinations for croup by year, excluding 1 DPEC.

## Abbreviations

DPECs	Dedicated pediatric emergency centers
ED	Emergency department
GECs	General emergency Centers
ICD-10	The International Classification of Diseases-10
NHAMCS	National Hospital Ambulatory Medical Care Survey
NHIS	National Health Insurance Service
NHSPIC	The National Health Screening Program for Infants and Children
