# Supplementary material for: Management Patterns of Croup in Korean Emergency Departments: A Nationwide Cohort Study
Source: Children (Basel). 2025 Sep 25;12(10):1301. doi: 10.3390/children12101301 (PMC12562596; doi:10.3390/children12101301)
Supplement: Supplementary file 1 [file children-12-01301-s001.zip › children-3850683-supplementary.pdf]

Supplemental Table S1. Steroids in detail.

N (%)	Overall (N=2223)	GECs (N=1932)	DPECs (N=291)	*P* Value
Dexamethasone	1050 (46.3)	896 (45.5)	154 (51.9)	0.081
Prednisolone	432 (19.1)	381 (19.3)	51 (17.2)	0.422
Hydrocortisone	55 (2.4)	49 (2.5)	6 (2.0)	0.777
Methylprednisolone	40 (1.8)	37 (1.9)	3 (1.0)	0.411

Values are presented as number (% of croup ED visits); GECs, general emergency centers; DPECs, dedicated pediatric emergency centers.

Supplemental Table S2. Changes in total steroid prescription by year in DPECs.

Hospital	2011	2012	2013	2014	2015
A*	45.5	63.2	76.5	81.8	100.0
B*	71.4	69.2	35.7	75.0	83.3
C*	100.0	90.9	66.7	100.0	0.0
D*	0.0	10.0	0.0	5.9	0.0
E*	85.7	85.7	80.0	58.3	83.3
F*	100.0	80.0	90.0	75.0	100.0
G†			40.0	100.0	100.0
H†			0.0	0.0	0.0
I†			40.0	77.8	50.0
J‡				87.5	100

Values are presented as number (% of croup ED visits for each year). Values are presented as number (% of croup ED visits for each year).GECs, general emergency centers; DPECs, dedicated pediatric emergency centers.

* Since 2011

† Since 2013

‡ Since 2014

Supplemental Table S3. Primary and secondary outcomes, excluding one DPEC.

N (%)	Overall (N=2177)	GECs (N=1932)	DPECs (N=245)	Risk difference (95% CI)	*P* Value
Total steroid prescription	1263 (58.0)	1087 (56.3)	176 (71.8)	15.5% (9.2–21.9)	<0.001
Nebulizer					
Epinephrine nebulizer	112 (5.1)	93 (4.8)	19 (7.8)	3.0% (0.0–6.5)	0.07
Salbutamol nebulizer	255 (11.7)	245 (12.7)	10 (4.1)	-8.6% (-12.3– -4.9)	<0.001
Radiographs					
Chest radiographs	1683 (77.3)	1520 (78.7)	163 (66.5)	-12.2% (-18.3– -6.1)	<0.001
Cervical spine radiographs	244 (11.2)	244 (12.6)	0 (0.0)	-12.6% (-15.0– -10.2)	<0.001
Sub-diagnosis respiratory disease					0.391
Viral pneumonia (J12)	1 (0.2)	1 (0.2)	0 (0.0)	-0.2% (-0.6– 0.2)	
Bacterial pneumonia (J15)	3 (0.5)	3 (0.6)	0 (0.0)	-0.6% (-1.4– 0.2)	
Pneumonia, unspecified (J18)	104 (17.7)	97 (18.9)	7 (9.3)	-9.6% (-15.2– -4.0)	
Acute bronchitis (J20)	301 (51.1)	260 (50.6)	41 (54.7)	4.1% (-5.1– 13.3)	
Acute bronchiolitis (J21)	179 (30.4)	152 (29.6)	27 (36.0)	6.4% (-2.9– 15.7)	
Acute lower respiratory infection, unspecified (J22)	1 (0.2)	1 (0.2)	0 (0.0)	-0.2% (-0.6– 0.2)	
Department					0.260
Emergency medicine	1111 (51.0)	1003 (51.9)	108 (44.1)	-7.8% (-15.0– -0.6)	
Pediatrics	1059 (48.6)	922 (47.7)	137 (55.9)	8.2% (0.8– 15.6)	
Surgery	4 (0.2)	4 (0.2)	0 (0.0)	-0.2% (-0.6– 0.2)	
Internal medicine	1 (0.0)	1 (0.1)	0 (0.0)	-0.1% (-0.3– 0.1)	
Otolaryngology	1 (0.0)	1 (0.1)	0 (0.0)	-0.1% (-0.3– 0.1)	
Family medicine	1 (0.0)	1 (0.1)	0 (0.0)	-0.1% (-0.3– 0.1)	
Disposition					
Discharge	1522 (69.9)	1315 (68.1)	207 (84.5)	16.4% (10.5–22.3)	<0.001

Values are presented as number (% of croup ED visits); ED, emergency department; GECs general emergency centers; DPECs, dedicated pediatric emergency centers.

Supplemental Table S4. Changes in ED prescription drugs and radiological examinations for croup by year, excluding one DPEC.

	**Variables**	**2008**	**2009**	**2010**	**2011**	**2012**	**2013**	**2014**	**2015**	***P* Value**
Overall	ED visits (N=2177)	16	74	264	265	476	472	394	216	
	Total steroid prescription	9 (56.2)	37 (50.0)	132 (50.0)	140 (52.8)	285 (59.9)	290 (61.4)	238 (60.4)	132 (61.1	0.020
	Dexamethasone	7 (43.8)	35 (47.3)	105 (39.8)	124 (46.8)	240 (50.4)	227 (48.1)	190 (48.2)	114 (52.8)	0.160
	Epinephrine nebulizer	1 (6.2)	5 (6.8)	10 (3.8)	9 (3.4)	14 (2.9)	33 (7.0)	23 (5.8)	17 (7.9)	0.039
	Salbutamol nebulizer	2 (12.5)	10 (13.5)	33 (12.5)	45 (17.0)	63 (13.2)	35 (7.4)	43 (10.9)	24 (11.1)	0.015
	Chest radiography	14 (87.5)	72 (97.3)	223 (84.5)	217 (81.9)	387 (81.3)	362 (76.7)	269 (68.3)	139 (64.4)	<0.001
	Cervical spine radiography	4 (25.0)	15 (20.3)	27 (10.2)	36 (13.6)	51 (10.7)	53 (11.2)	46 (11.7)	12 (5.6)	0.012
GECs	ED visits (N=1932)	16	74	264	236	416	399	338	189	
	Total steroid prescription	9 (56.2)	37 (50.0)	132 (50.0)	120 (50.8)	240 (57.7)	243 (60.9)	196 (58.0)	110 (58.2)	0.079
	Dexamethasone	7 (43.8)	35 (47.3)	105 (39.8)	109 (46.2)	204 (49.0)	185 (46.4)	153 (45.3)	95 (50.3)	0.409
	Epinephrine nebulizer	1 (6.2)	5 (6.8)	10 (3.8)	8 (3.4)	10 (2.4)	22 (5.5)	21 (6.2)	16 (8.5)	0.036
	Salbutamol nebulizer	2 (12.5)	10 (13.5)	33 (12.5)	44 (18.6)	62 (14.9)	32 (8.0)	40 (11.8)	22 (11.6)	0.013
	Chest radiography	14 (87.5)	72 (97.3)	223 (84.5)	191 (80.9)	345 (82.9)	308 (77.2)	240 (71.0)	127(67.2)	<0.001
	Cervical spine radiography	4 (25.0)	15 (20.3)	27 (10.2)	36 (15.3)	51 (12.3)	53 (13.3)	46 (13.6)	12 (6.3)	0.023
DPECs	ED visits (N=245)	N/A	N/A	N/A	29	60	73	56	27	
	Total steroid prescription	N/A	N/A	N/A	20 (69.0)	45 (75.0)	47 (64.4)	42 (75.0)	22 (81.5)	0.415
	Dexamethasone	N/A	N/A	N/A	15 (51.7)	36 (60.0)	42 (57.5)	37 (66.1)	19 (70.4)	0.552
	Epinephrine nebulizer	N/A	N/A	N/A	1 (3.4)	4 (6.7)	11 (15.1)	2 (3.6)	1 (3.7)	0.081
	Salbutamol nebulizer	N/A	N/A	N/A	1 (3.4)	1 (1.7)	3 (4.1)	3 (5.4)	2 (7.4)	0.751
	Chest radiography	N/A	N/A	N/A	26 (89.7)	42 (70.0)	54 (74.0)	29 (51.8)	12 (44.4)	<0.001
	Cervical spine radiography	N/A	N/A	N/A	0 (0.0)	0 (0.0)	0 (0.0)	0 (0.0)	0 (0.0)	

Values are presented as number (% of croup ED visits for each year). ED, emergency department; GECs, general emergency centers; DPECs, dedicated pediatric emergency centers; N/A, not applicable (not yet designated as DPEC).
